# Estimating the Relative Crystallinity of Biodegradable Polylactic Acid and Polyglycolide Polymer Composites by Machine Learning Methodologies

**DOI:** 10.3390/polym14030527

**Published:** 2022-01-28

**Authors:** Jing Wang, Mohamed Arselene Ayari, Amith Khandakar, Muhammad E. H. Chowdhury, Sm Ashfaq Uz Zaman, Tawsifur Rahman, Behzad Vaferi

**Affiliations:** 1 College of Energy Engineering, Yulin University, Yulin 719000, China; 2 Department of Civil and Architectural Engineering, College of Engineering, Qatar University, Doha 2713, Qatar; 3 Technology Innovation and Engineering Education, College of Engineering, Qatar University, Doha 2713, Qatar; 4 Electrical Engineering Department, College of Engineering, Qatar University, Doha 2713, Qatar; amitk@qu.edu.qa (A.K.); mchowdhury@qu.edu.qa (M.E.H.C.); tawsifur.rahman@qu.edu.qa (T.R.); 5 Department of Information Science & Technology, Universiti Kebangsaan Malaysia, Bangi 43600, Malaysia; ashfaquzzaman2@gmail.com; 6 Department of Chemical Engineering, Shiraz Branch, Islamic Azad University, Shiraz 7473171987, Iran; behzad.vaferi@gmail.com

**Keywords:** polylactic acid, polyglycolide, biodegradable composite, relative crystallinity, machine learning methods

## Abstract

Biodegradable polymers have recently found significant applications in pharmaceutics processing and drug release/delivery. Composites based on poly (L-lactic acid) (PLLA) have been suggested to enhance the crystallization rate and relative crystallinity of pure PLLA polymers. Despite the large amount of experimental research that has taken place to date, the theoretical aspects of relative crystallinity have not been comprehensively investigated. Therefore, this research uses machine learning methods to estimate the relative crystallinity of biodegradable PLLA/PGA (polyglycolide) composites. Six different artificial intelligent classes were employed to estimate the relative crystallinity of PLLA/PGA polymer composites as a function of crystallization time, temperature, and PGA content. Cumulatively, 1510 machine learning topologies, including 200 multilayer perceptron neural networks, 200 cascade feedforward neural networks (CFFNN), 160 recurrent neural networks, 800 adaptive neuro-fuzzy inference systems, and 150 least-squares support vector regressions, were developed, and their prediction accuracy compared. The modeling results show that a single hidden layer CFFNN with 9 neurons is the most accurate method for estimating 431 experimentally measured datasets. This model predicts an experimental database with an average absolute percentage difference of 8.84%, root mean squared errors of 4.67%, and correlation coefficient (R^2^) of 0.999008. The modeling results and relevancy studies show that relative crystallinity increases based on the PGA content and crystallization time. Furthermore, the effect of temperature on relative crystallinity is too complex to be easily explained.

## 1. Introduction

Biodegradable materials have recently grown in popularity due to their wide applicability in different practices, including for clinical purposes [1,2,3], drug delivery [4,5], and waste remediation [6,7,8]. Poly (L-lactic acid) (PLLA) is a low-toxic biodegradable polymer with good mechanical properties. Although PLLA has relatively satisfactory mechanical characteristics, its crystallization rate is slow. Hence, the fabrication of composites [9] based on PLLA with a better crystallization behavior has been suggested [10,11,12]. Chen et al. synthesized several completely biodegradable PLLA-based composites by dispersing polyglycolide (PGA) fibers in the PLLA body [12]. Several laboratory-scale investigations have been carried out to examine the effect of crystallization time, temperature, and PGA fiber dosage on the relative crystallinity of pure PLLA and PLLA/PGA composites [12].

Generally, the application domain of polymers is directly related to their physicochemical properties, including their tensile strength, elasticity, glass transition temperature, solubility, and crystallinity [12]. Crystallization is among the most complicated and longest-standing challenges related to polymer [13,14,15], composite [16], and zeolite [17] research and development. The relative crystallinity of polymers shows the degree of alignment of polymeric chains to one another [18]. Balani et al. claimed that polymer strength increases by increasing its crystallinity [19]. They also introduced the significant intermolecular bonding of the crystalline phase as the main factor responsible for their observation [19].

Differential scanning calorimetry, Raman spectroscopy, X-ray diffraction, nuclear magnetic resonance, infrared spectroscopy, small-angle X-ray scattering, and microscopy are the most common techniques for determining crystallinity and crystallization behavior [20]. Despite the diversity of experimental techniques, theoretical and modeling approaches are still rare in this topic. Molecular simulation is the most utilized method that provides some insight into polymer crystallization [21]. Therefore, our understanding of this complex subject is still incomplete and deserves further analysis.

The current study utilizes machine learning methods to accurately estimate PLLA/PGA composites’ relative crystallinity. Six different artificially intelligent categories, including four artificial neural networks (ANN), two adaptive neuro-fuzzy inference systems, and least-squares support vector regression, were considered for this task. Systematic comparison analyses using four statistical indices confirmed that the cascade feedforward neural network provides the most reliable estimations for the relative crystallinity of PLLA/PGA composites. This model accurately predicts 431 experimentally measured datasets with an impressive average absolute percentage difference of 8.84%, root mean squared errors of 4.67%, and correlation coefficient (R^2^) of 0.999008. This model confirms that the relative crystallinity of pure PLLA and PLLA/PGA composites increases by increasing the crystallization time and reduces by reducing the crystallization temperature. Furthermore, PGA content in the PLLA-based composite has a weak increasing effect on relative crystallinity. To the best of our knowledge, there are no other studies in the literature which have conducted intelligent modeling related to the crystallinity processes of biodegradable PLLA/PGA composites.

## 2. Materials and Experiments

Chen et al. utilized the solvent method to fabricate several PLLA/PGA composites with 2–8 weight percent (wt%) of the PGA fiber [12]. The differential scanning calorimetry (DSC) technique was employed to study the effect of PGA fibers on the isothermal crystallization of fabricated composites [12]. Table 1 shows the range of conducted experiments to measure relative crystallinity as a function of time, temperature, and PGA dosage in the considered polymers.

It should be mentioned that pure PLLA is considered a composite with zero wt% of PGA fibers. This table shows that PLLA and all PLLA/PGA composites experience a maximum relative crystallinity of 100%. On the other hand, the crystallization time of pure PLLA is almost twice that of a composite with 8 wt% of PGA fibers.

**Figure 1:**
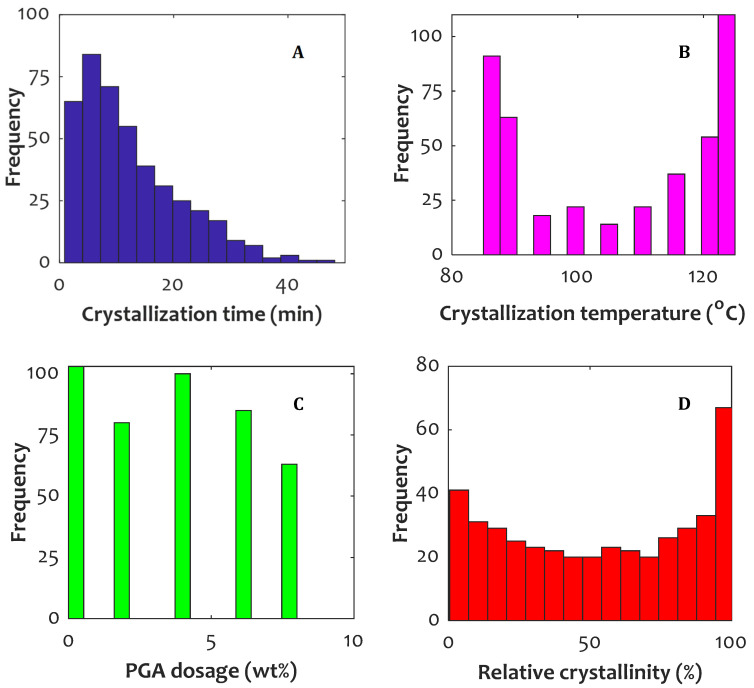
Histogram of experimental measurements for all of crystallization times (**A**), crystallization temperatures (**B**), PGA contents (**C**), and relative crystallinities (**D**).

For a better presentation of the experimental study conducted by Chen et al. [12], the histogram of the considered variables is plotted in Figure 1. Histograms of crystallization time, temperature, PGA content of composites, and relative crystallinity are depicted in Figure 1A–D, respectively.

## 3. Methodology

Machine learning is a trusted method to accurately estimate behaviors of different phenomena ranging from disease identification [22,23,24,25,26] and privacy-preserving healthcare [27,28] to sustainable development [29]. As previously noted, this study constructs different machine learning methods and compares their accuracies to identify the most trustworthy topology for calculating the relative crystallinity of pure PLLA and PLLA/PGA composites. The multilayer perceptron neural network (MLPNN), recurrent neural network (RNN), cascade feedforward neural network (CFFNN), adaptive neuro-fuzzy inference system with subtractive clustering (ANFIS2) and c-means clustering (ANFIS3) membership functions, and least-squares support vector regression (LSSVR) have been employed in this regard.

### 3.1. Artificial Neural Networks

Artificial neural networks were originally inspired by the operating practice of the neurological system of human beings [30]. Neurons are the smallest meaningful parts of neurological and artificial neural networks [31]. It is possible to place several neurons in some successive layers to create different topologies of the ANN. The MLPNN [32], CFFNN [33], RNN [34], radial basis function neural networks, and general regression neural networks are the most well-known ANN types in this regard. Our literature review confirmed that the first three aforementioned models often provide acceptable accuracy for regression-based problems. The mathematical and working backgrounds of MLPNN [35], CFFNN, and RNN [36] are well presented in the literature.

Cybenko theoretically confirmed that ANN models with only one hidden layer equipped with nonlinear, continuous, and differentiable activation functions are able to simulate even the most complicated phenomena [37]. The hyperbolic tangent and logistic activation functions satisfy the conditions proposed by Cybenko [37]. Therefore, it is only necessary to determine the number of neurons placed in the hidden layer.

### 3.2. Adaptive Neuro-Fuzzy Inference Systems

Adaptive neuro-fuzzy inference systems can be imagined as an organized combination of fuzzy logic and ANN methodologies [38]. This type of machine learning category is often built using five interconnected layers [38]. The membership function is the central part of the working procedure of the ANFIS-based model. Subtractive clustering and c-means clustering are the two most widely used membership functions in the ANFIS structure. It is necessary to determine the cluster radius for the former and the number of clusters for the latter [38]. Furthermore, an appropriate training algorithm also needs to be appropriately determined for developing the ANFIS-based model [38].

### 3.3. Least-Squares Support Vector Regression

Least-squares support vector regression is another machine learning method used in the current study [39]. This intelligent scenario uses the kernel function to transform the independent variable into a multidimensional space. Then, it is possible to linearly relate a target to its transformed independent variables. Suykens et al. comprehensively explained both the mathematical background and working procedure of the LSSVR [40]. An appropriate type of kernel function should be determined for the LSSVR-based estimator. Linear, polynomial, and Gaussian are possible kernel functions for incorporation in the LSSVR structure [40].

## 4. Results and Discussion

### 4.1. Relevancy Analyses

Both experimental [41] and modeling [42] studies have investigated the effects of the main influential variables on the considered dependent/target variable. Some statistical-based methods are available for quantizing the direction and magnitude of relevancy between any pair of dependent–independent variables [43,44]. Spearman [45], Pearson [46], and Kendall [47] are three main instances in this field. These methods provide an index between −1 and +1 to show the direction and magnitude of dependency of a target to its influential features [48]. Table 2 explains the physical meaning of outcomes of these relevancy-monitoring methods.

In summary, the negative domain shows the indirect dependency of a dependent variable to an independent one and vice versa. On the other hand, −1 and +1 are associated with the strongest indirect and direct relationships, respectively. The magnitude of this relevancy decreases by converging the index to zero.

**Figure 2:**
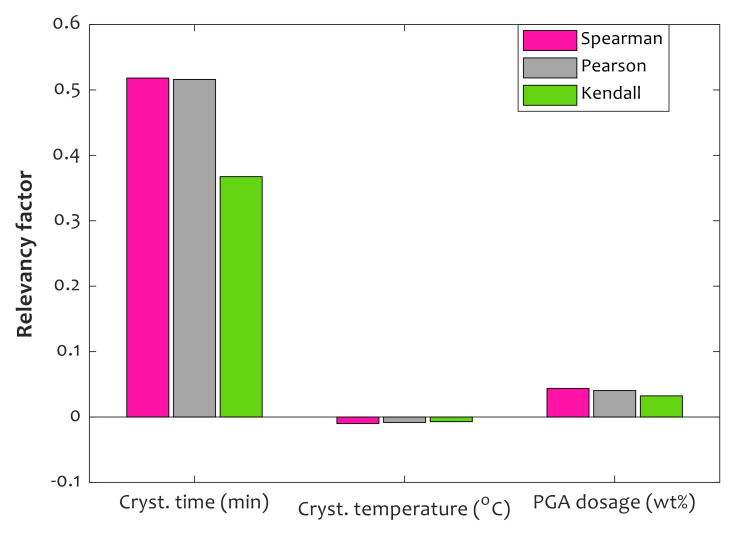
Interdependency of relative crystallinity on time, temperature, and PGA dosage.

The results of applying the aforementioned relevancy scenarios on the collected databank for the relative crystallinity of the PLLA/PGA composites are graphically presented in Figure 2. Relative crystallinity directly relates to the crystallization time (strong) and PGA content of composites (weak). On the other hand, relative crystallinity has weak indirect relevancy with crystallization temperature.

### 4.2. Developing Machine Learning Methods

In order to efficiently use the considered machine learning techniques, their topologies need to be appropriately determined [37,49,50]. Since several rules of thumb prespecify some structural features of CFFNN, MLPNN, RNN, LSSVR, ANFIS2, and ANFIS3, it is only necessary to determine the rest of the features using a trial-and-error procedure. Table 3 divides the structural features of each technique into fixed and adjustable ones.

### 4.3. Selecting the Best Topology for Machine Learning Methods

The experimental databank of the relative crystallinity of PLLA/PGA composites is randomly divided into training and testing collections. The former includes 366 datasets (85%), and the latter constates 65 measurements (15%). Five-fold cross-validation utilizes the training collection to determine the adjustable structural features and hyperparameters of the considered machine learning techniques. The testing collection is then engaged in evaluating the performance of the constructed paradigms. Four statistical-based accuracy indices, including average absolute percentage difference (*AAPD*%), root mean squared errors (*RMSE*), correlation coefficient (*R*^2^), and relative absolute percentage error (*RAPE*%), help to find the most reliable topology for each machine learning technique. Equations (1)–(4) define mathematical formulations of *AAPD*%, *RMSE*, *R*^2^, and *RAPE*%, respectively.

(1) $$AAPD\%=\frac{100}{N}\sum_{j=1}^{N}\frac{{\left|RC^{\exp}-RC^{cal}\right|}_{j}}{RC_{j}^{\exp}}$$

(2) $$RMSE=\sqrt{\frac{1}{N}\;{\sum_{j=1}^{N}{\left(RC^{\exp}-RC^{cal}\right)}_{j}}^{2}}$$

(3) $$R^{2}=1-\;\frac{\sum_{j=1}^{N}{\left(RC^{\exp}-RC^{cal}\right)}_{j}{}^{2}}{\sum_{j=1}^{N}{\left(RC^{\exp}-\bar{RC^{\exp}}\right)}_{j}{}^{2}}$$

(4) $$RAPE\%=\;\frac{100\;\times \sum_{j=1}^{N}{\left|RC^{\exp}-RC^{cal}\right|}_{j}}{\sum_{j=1}^{N}{\left|RC^{\exp}-\bar{RC^{\exp}}\right|}_{j}}$$

All above equations need experimental measurements ($RC^{\exp}$), calculated values ($RC^{cal}$) of the relative crystallinity (*RC*), and numbers of training or testing datasets (*N*) to be calculated.

The performance of the constructed models was compared using these statistical criteria to find those adjustable features that present the highest accuracy in the training and testing stages. Table 4 introduces the best adjustable features for each class of the machine learning method. This table also reports the accuracy of the selected models for the training and testing collections as well as their combination, i.e., overall database. The reported accuracies in Table 4 show that the adaptive neuro-fuzzy inference system with the c-means clustering membership function (i.e., ANFIS3) is the model that predicts both training and testing collections with the highest uncertainty. This model estimates 431 experimental measurements of the relative crystallinity of PLLA/PGA composites with *AAPD* = 24.78%, *RAPE* = 14.53%, *RMSE* = 6.54, and *R*^2^ = 0.980306.

**Figure 3:**
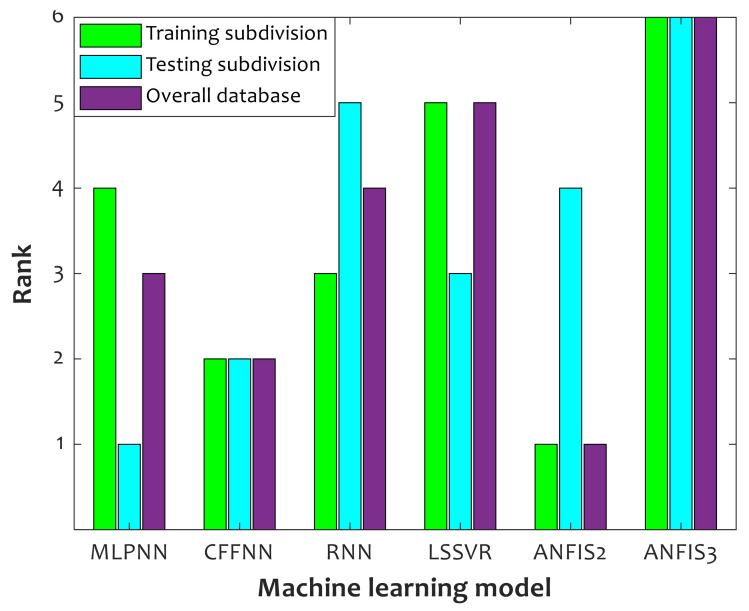
Ranking of machine learning methods during model development, model validation, and their combination.

Figure 3 reports the outcome of the ranking analysis performed to order the selected models in Table 4 based on their average prediction accuracy over the training, testing, and overall collections. Indeed, the average efficiency of each model has been measured using their *AAPD*%, *RAPE*%, *RMSE*, and *R*^2^ values. This figure shows that ANFIS2 and MLPNN have the best performance in the training and testing stage, respectively. Since MLPNN badly estimates the training collection and ANFIS2 prediction for the testing stage is not very good, neither of them should be selected as the most trusted model. On the other hand, the CFFNN model with the second prediction ranking for the training, testing, and overall collections is a better selection for estimating the relative crystallinity of pure PLLA and PLLA/PGA composites.

### 4.4. Investigating the Effect of Activation Function on CFFNN Performances

Cybenko stated that a continuous, nonlinear, and differentiable activation function such as hyperbolic tangent and logistic is better to utilize in the structure of artificial neural networks [37]. However, it is not clear what combination of these activation functions shows the best predictive performance. Table 5 reports the prediction accuracy of CFFNN with different combinations of the hyperbolic tangent and logistic activation functions. The first row of this table shows the previously achieved results (see Table 4) by the hyperbolic tangent and logistic activation functions in the hidden and output layers, respectively. The second row of Table 5 confirms that it is possible to improve the prediction accuracy of the CFFNN model.

In summary, a single hidden layer CFFNN with nine hidden neurons equipped with the logistic activation functions in its layers is the most accurate model for predicting the relative crystallinity of pure PLLA and PLLA/PGA composites. Therefore, all the following analyses were directed using this intelligent method.

**Figure 4:**
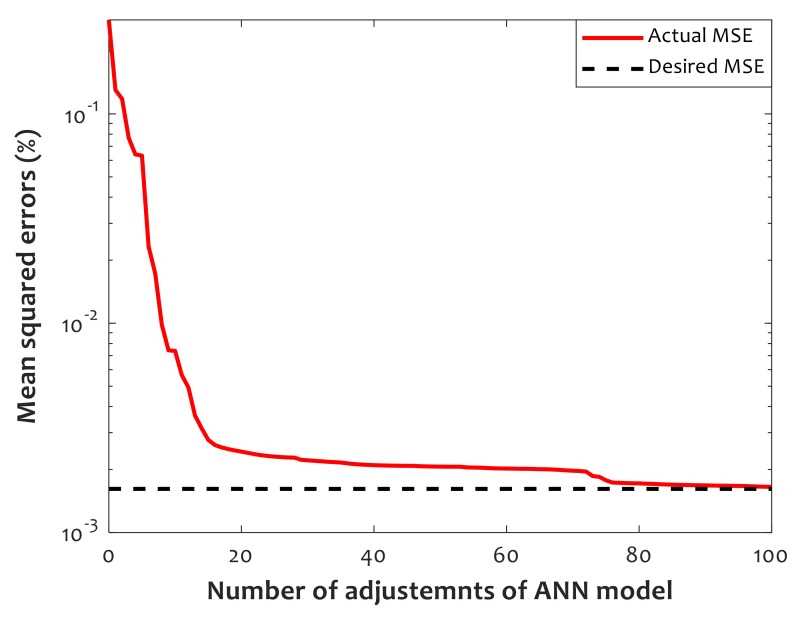
Results of the iterative procedure conducted using the Levenberg–Marquardt to train the CFFNN method.

Figure 4 presents the iterative procedure that the Levenberg–Marquardt passes to adjust hyperparameters of the CFFNN model. After 100 iterations, the mean squared errors (*MSE*) between experimental and prediction values of the relative crystallinity converge to the predefined desired value, i.e., *MSE* = 0.75 × 10^−3^. The *MSE* value can be calculated using Equation (5).

(5) $$MSE=\frac{1}{N}\;{\sum_{j=1}^{N}\left(RC^{\exp}-RC^{cal}\right)}_{j}^{2}$$

### 4.5. Analyzing the Performance of the CFFNN Model

**Figure 5:**
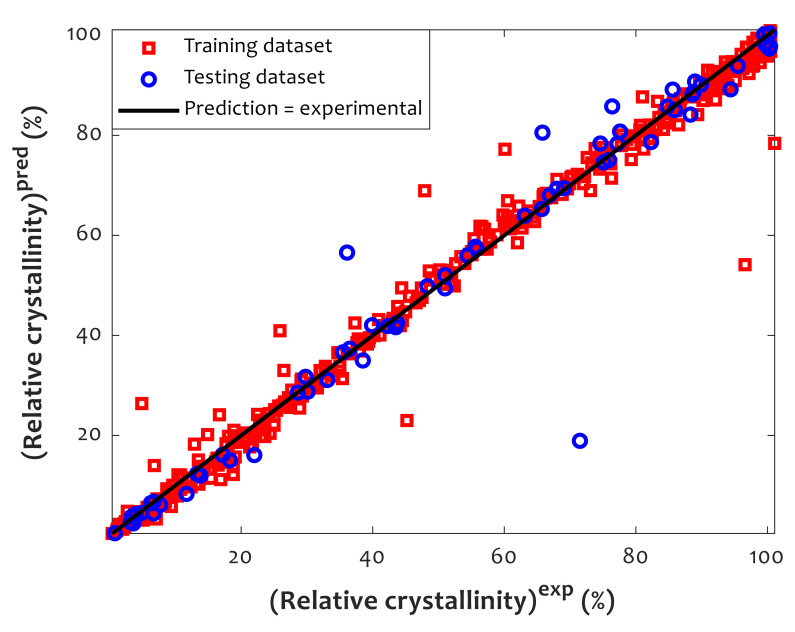
Predicted versus actual measured values of relative crystallinity.

A cross-plot of the estimated relative crystallinities by the proposed CFFNN with respect to their corresponding experimentally measured information is shown in Figure 5. It can be observed that almost all CFFNN predictions have been successfully mapped on their associated experimentally measured data points. Moreover, the previously achieved results in Table 4 state that the regression coefficients for the training, testing, and overall collections are 0.990058, 0.990337, and 0.990082, respectively.

**Figure 6:**
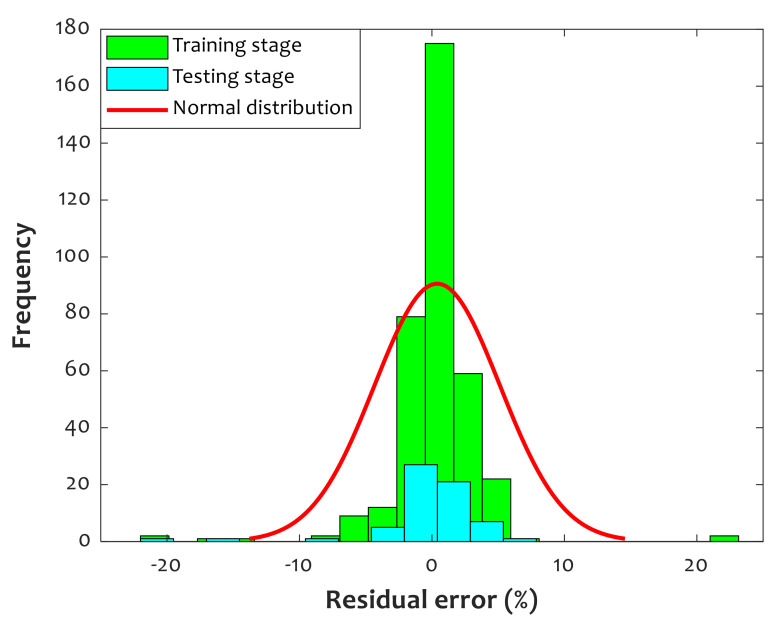
Histogram presentation of deviation between predicted and actual values of relative crystallinity (average error = 0.398%, standard deviation = 4.72%).

Figure 6 depicts the residual error (Equation (6)) histogram between the CFFNN predictions and actual values of relative crystallinity of pure PLLA and PLLA/PGA composites.

(6) $$\mathrm{Residual}\;\mathrm{error}=\left(RC^{\exp}-RC^{cal}\right)$$

This figure confirms that the maximum residual error of +10% and minimum value of −10% are provided by the fabricated CFFNN model. It can also be observed that ~175 training samples and ~23 testing samples were simulated with zero residual error. The fitted red curve confirms that the observed results obey normal distribution.

**Figure 7:**
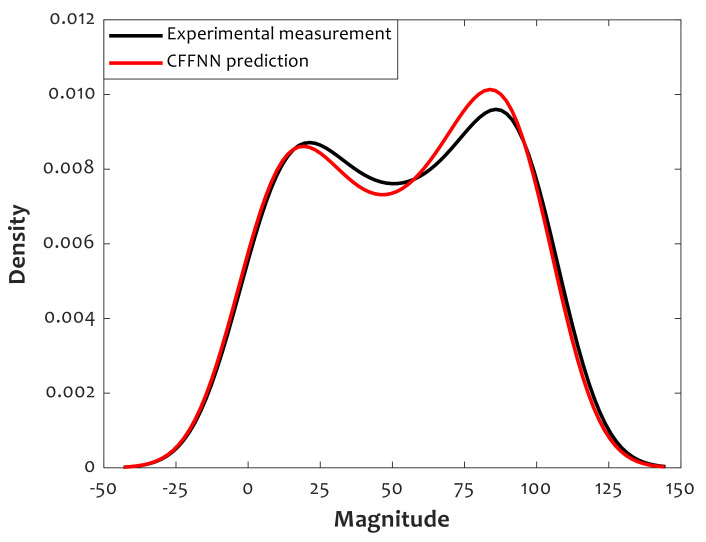
Kernel density estimation for actual measurements and CFFNN predictions.

Kernel density estimation [56] is employed to plot a distribution of the CFFNN predictions and actual values of the relative crystallinity data collections (see Figure 7). Although the data distribution is very close to normal distribution, two normal distributions can be simply detected. Furthermore, the distributions of CFFNN predictions and actual values are almost identical. The predicted and actual distributions are slightly different between the magnitudes of 15 and 85. Hence, Figure 7 confirms the robustness of the proposed CFFNN model.

### 4.6. Checking the Validity of Experimental Data

**Figure 8:**
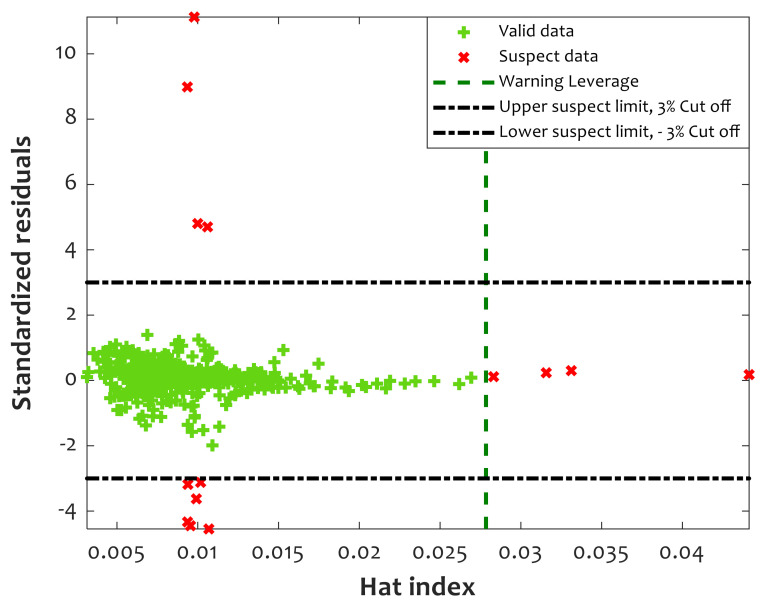
Applying leverage analysis to detect reliable as well as outlier information.

Since CFFNN has been constructed using experimental measurements of the relative crystallinity of PLLA/PGA composites, its reliability may be affected by potential outliers in the collected databank [51]. Therefore, it is a good idea to evaluate the level of poisoning of the experimental databank by such outliers [51]. The leverage is a practical statistical method for distinguishing valid and suspect measurements in a given database [51]. This method identifies valid/suspect data by plotting the standardized residual against the hat index (see Figure 8). The mathematical form of the standardized residual (*SR*) is shown using Equation (7).

(7) $$SR\;=\;{\left(\frac{RD}{SD}\right)}_{k}\;k=1,\;2,\;\ldots ,N$$

A region bounded by −3 < standardized residual < +3 and Hat index < warning leverage is valid, and all five other parts are suspect domains. Based on Equation (8), the numbers of influential factors (*IF*) and experimental data (*N*) are required to calculate the warning leverage (*WL*). Since the current study utilizes three influential factors (i.e., crystallization time, crystallization temperature, and PGA dosage) to estimate relative crystallinity (*N* = 431), *WL* = 0.0278 (Figure 8: vertical dashed green line) [39].

(8) $$WL\;=\;3\;\times \;\left(IF\;+\;1\right)/N$$

The outcomes of applying the leverage method on the PLLA/PGA crystallization database are depicted in Figure 8. It can be seen that 417 datasets are valid, and only 14 measurements may be outliers. Applying the leverage method to the experimental database demonstrates that more than 97% are valid measurements. Therefore, the validity of the experimental data is approved, and the engineered CFFNN method is ready to be used in real applications.

### 4.7. Monitoring the Effect of Influential Features on Relative Crystallinity

**Figure 9:**
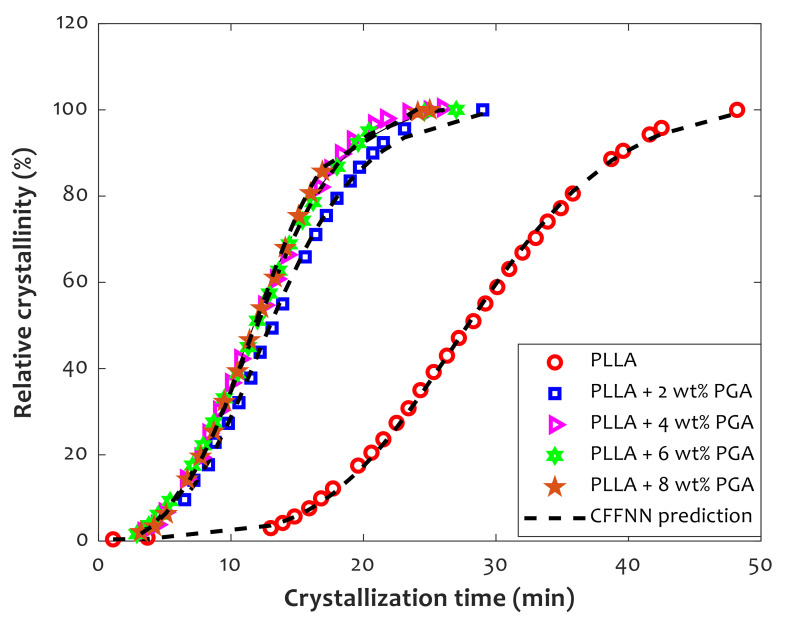
The effect of PGA dosage and crystallization time on rate of crystallization at 125 °C.

Figure 9 utilizes experimentally measured information as well as CFFNN prediction to investigate the effect of time and mass dosage of PGA fibers on the relative crystallinity of pure PLLA and its composites. An excellent compatibility level exists between actual and predicted crystallinity information. Experimental observations, as well as modeling results, show that the relative crystallinity of pure PLLA and PLLA/PGA gradually increases with increasing time. Furthermore, increasing the PGA mass dosage from 0% to 8% decreases the time needed to reach the maximum relative crystallinity of 100%. It can be seen that pure PLLA experiences maximum crystallinity at 50 min, while the PLLA/PGA composite reaches the maximum value after just 30 min. It can be concluded that the addition of PGA fibers to the PLLA structure improves the crystallization rate. However, all composites reach maximum crystallinity in half the time that is required for pure PLLA.

**Figure 10:**
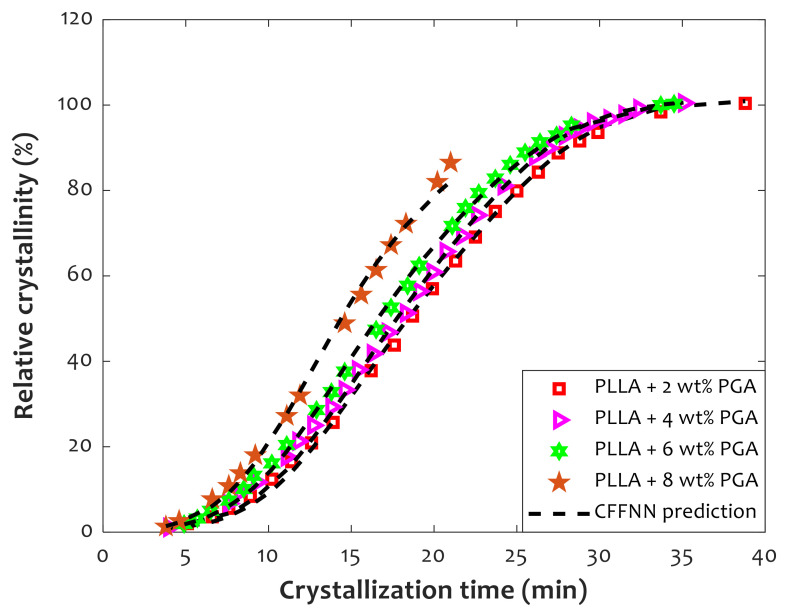
How PGA dosage affects relative crystallization of PLLA/PGA composites at 85 °C.

The effect of PGA fiber dosage and crystallization time on the relative crystallinity of PLLA/PGA composites is shown in Figure 10. This figure shows that the required time for achieving full crystallization decreases by increasing the PGA content in the composite structure. The composite containing 8 weight percent of PGA fibers reaches maximum crystallinity faster than the other available PLLA/PGA composites.

**Figure 11:**
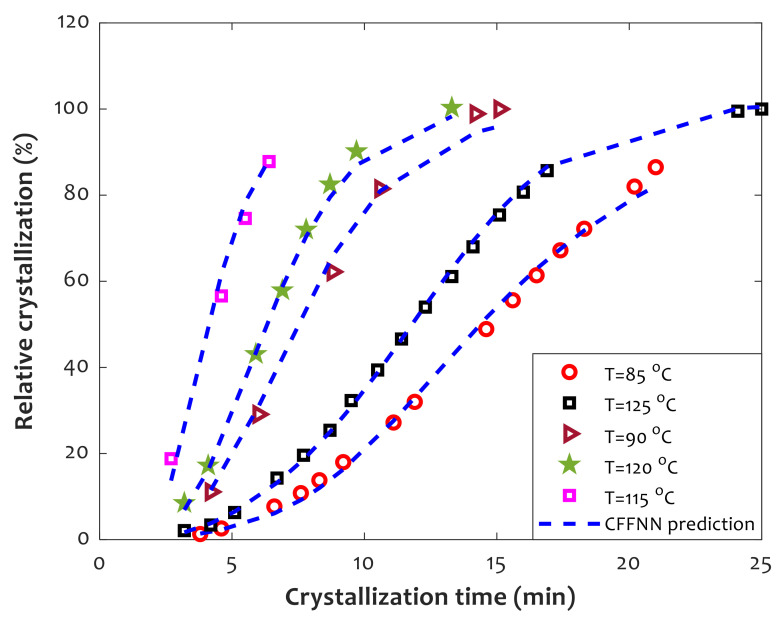
Isothermal relative crystallinity of the PLLA/PGA composite with 8 wt% of the fiber.

The effect of temperature on the relative crystallinity of PLLA-based composites containing 8 wt% of PGA fibers is presented in Figure 11. It can easily be seen that relative crystallinity shows a complex reaction to temperature change. Despite this complex behavior, CFFNN successfully predicted crystallinity variation and estimated all individual experimental samples.

Relative crystallization from below the melting temperature to above glass temperature often shows a complex behavior [13]. This complex behavior is observed in long-chain polymers and also monomeric substances [13]. At the vicinity of melting temperature, the crystallization rate is very slow [13]. As the temperature further decreases, the crystallization rate gradually increases and finally reaches its maximum value [13]. At temperatures below this maximum condition, the overall crystallization rate is retarded once again [13].

### 4.8. Transferability of the Proposed Model

All empirical, semi-empirical, or intelligent methodologies extracted from historical data are only applicable for interpolation purposes on a considered system. Indeed, their extrapolation ability is so limited that they should be used with caution. Therefore, the deployed cascade feedforward neural network in this study can only be applied to estimate the relative crystallinity of biodegradable polylactic acid and polyglycolide polymer composites covering the reported values in Table 1. There is no guarantee of accurately estimating the relative crystallinity of other polymer composites using the constructed CFFNN machine.

## 5. Conclusions

This study used six different machine learning categories to correlate the relative crystallinity of pure PLLA and PLLA/PGA composites to crystallization time, crystallization temperature, and PGA dosage in composites. So many intelligent models have been constructed, and their accuracy has been compared to choose the best one for the given purpose. The ranking study using four accuracy indices confirmed that the cascade feedforward neural network has the highest level of agreement with the 431 experimentally measured datasets. This model predicted the available databank with an extraordinary correlation coefficient (*R*^2^) of 0.999008, root mean squared errors of 4.67%, and average absolute percentage difference of 8.84%. Reliability checking confirmed that 97% of the experimental information is valid. The results also showed that relative crystallinity directly relates to crystallization time and PGA dosage in the composites, and it has a weak indirect relationship with crystallization temperature. Indeed, relative crystallinity increases by increasing time and PGA dosage in the composites. On the other hand, variation of relative crystallinity based on temperature is too complex to suggest a general route for its behavior. The literature has also observed such complex behavior for both long-chain polymers and monomeric substances.

## Figures and Tables

**Table 1:** Experiment data for the relative crystallinity of PLLA/PGA composites [12].

Crystallization Time (min)	Crystallization Temperature (°C)	PGA Dosage (wt%)	Relative Crystallinity (%)	Numbers of Measurements
0–50	90–125	0	0–100	103
0–40	85–125	2	0–100	80
0–35	85–125	4	0–100	100
0–35	85–125	6	0–100	85
0–25	85–125	8	0–100	63

**Table 2:** Physical meaning of the Spearman, Pearson, and Kendall indices.

Index Value	Direction of Relevancy	Magnitude of Relevancy
−1 to < 0	Indirect	Magnitude of indirect relationship increases from zero to −1
0	No dependency	No dependency
<0 to +1	Direct	Magnitude of direct relationship increases from zero to 1

**Table 3:** Summary of the trial-and-error process to find the best structural features of the machine learning methods.

Machine Learning Method	Structural Property		Numbers of Model
	Fixed Property	Adjustable Property	
MLPNN	Number of hidden layers, i.e., two [37] The activation function of the hidden layer, i.e., hyperbolic tangent [37] The activation function of the hidden layer, i.e., logistic [37] Training algorithm, i.e., Levenberg–Marquardt [51]	Number of hidden neurons	200
CFFNN	Number of hidden layers, i.e., two [37] The activation function of the hidden layer, i.e., hyperbolic tangent [37] The activation function of the hidden layer, i.e., logistic [37] Training algorithm, i.e., Levenberg–Marquardt [51]	Number of hidden neurons	200
RNN	Number of hidden layers, i.e., two [37] The activation function of the hidden layer, i.e., hyperbolic tangent [37] The activation function of the hidden layer, i.e., logistic [37] Training algorithm, i.e., scaled conjugate gradient [52]	Number of hidden neurons	160
LSSVR	Training algorithm, i.e., least-squares method [40]	Kernel function	150
ANFIS2	Membership function, i.e., subtractive clustering [38,53]	Radius of cluster Training algorithm	400
ANFIS3	Membership function, i.e., c-means clustering [54,55]	Number of clusters Training algorithm	400

**Table 4:** The most appropriate features for the machine learning methods determined through the trial-and-error process.

Model	The Most Appropriate Characteristics	Collection	AAPD%	RAPE%	RMSE	R^2^
MLPNN	Nine hidden neurons	Training	11.13	7.38	4.95	0.988679
	Hyperbolic tangent and logistic	Testing	6.25	5.37	2.38	0.997467
	Levenberg optimization algorithm	Overall	10.39	7.07	4.65	0.990062
CFFNN	Nine hidden neurons	Training	8.74	6.68	4.54	0.990058
	Hyperbolic tangent and logistic	Testing	9.42	7.28	5.32	0.990337
	Levenberg optimization algorithm	Overall	8.84	6.76	4.67	0.990082
RNN	Seven hidden neurons	Training	10.92	9.81	4.00	0.992677
	Hyperbolic tangent and logistic	Testing	11.07	13.76	9.14	0.966081
	Scaled conjugate gradient algorithm	Overall	10.94	10.44	5.12	0.988174
LSSVR	Gaussian kernel function	Training	13.03	8.14	5.22	0.987382
		Testing	14.13	8.78	4.33	0.992005
		Overall	13.20	8.24	5.09	0.988064
ANFIS2	Hybrid optimization algorithm Cluster radius = 0.5	Training	8.54	5.27	4.41	0.991163
		Testing	16.28	8.79	5.36	0.985432
		Overall	9.71	5.74	4.57	0.990414
ANFIS3	Hybrid optimization algorithm Nine clusters	Training	25.81	13.87	6.29	0.981923
		Testing	19.01	18.39	7.78	0.971648
		Overall	24.78	14.53	6.54	0.980306

**Table 5:** Investigating the effect of activation functions on the predictive performances of the CFFNN method.

Hidden Layer	Output Layer	Training	Testing	Overall
Hyperbolic tangent	Logistic	8.74	9.42	8.84
Logistic	Logistic	7.97	8.61	8.06
Logistic	Hyperbolic tangent	8.33	6.80	8.10
Hyperbolic tangent	Hyperbolic tangent	9.35	5.53	8.78

## Data Availability

The data and MATLAB source codes have been provided as supplementary files.

## Supplementary Materials

The following supporting information can be downloaded at: https://www.mdpi.com/article/10.3390/polym14030527/s1, Data and MATLAB source codes.
